# Information-Theoretic Analysis of a Family of Improper Discrete Constellations

**DOI:** 10.3390/e20010045

**Published:** 2018-01-11

**Authors:** Ignacio Santamaria, Pedro M. Crespo, Christian Lameiro, Peter J. Schreier

**Affiliations:** 1 Departamento de Ingeniería de Comunicaciones, Universidad de Cantabria, 39005 Santander, Spain; 2 TECNUN, Universidad de Navarra, 20018 San Sebastián, Spain; 3 Signal & System Theory Group, Paderborn University, 33098 Paderborn, Germany

**Keywords:** improper signals, mutual information, Gaussian noise channels, discrete constellations

## Abstract

Non-circular or improper Gaussian signaling has proven beneficial in several interference-limited wireless networks. However, all implementable coding schemes are based on finite discrete constellations rather than Gaussian signals. In this paper, we propose a new family of improper constellations generated by widely linear processing of a square *M*-QAM (quadrature amplitude modulation) signal. This family of discrete constellations is parameterized by κ, the circularity coefficient and a phase ϕ. For uncoded communication systems, this phase should be optimized as $\phi^{*}\left(\kappa \right)$ to maximize the minimum Euclidean distance between points of the improper constellation, therefore minimizing the bit error rate (BER). For the more relevant case of coded communications, where the coded symbols are constrained to be in this family of improper constellations using $\phi^{*}\left(\kappa \right)$, it is shown theoretically and further corroborated by simulations that, except for a shaping loss of 1.53 dB encountered at a high signal-to-noise ratio (snr), there is no rate loss with respect to the improper Gaussian capacity. In this sense, the proposed family of constellations can be viewed as the improper counterpart of the standard proper *M*-QAM constellations widely used in coded communication systems.

## 1. Introduction

In the theoretical analysis of wireless communication systems, the transmitted symbols are typically assumed to be proper complex Gaussian random variables, i.e., the transmitted symbols are complex Gaussian and uncorrelated with their complex conjugate. This is motivated by the fact that such an input distribution achieves the capacity of the point-to-point, broadcast and multiple-access channels [1]. In more complex interference-limited scenarios, such as the interference channel, transmitting proper Gaussian signals is in general suboptimal, so assuming this input distribution in such cases is not theoretically justified. As a matter of fact, recent works have shown that, when treating interference as noise, a higher transmitted rate may be achieved in interference-limited networks by transmitting improper Gaussian signals, which are correlated with their complex conjugate [2,3,4,5,6,7,8].

Improper signals are common in communications since many important digital modulation schemes yield improper signals. Examples are binary phase shift keying (BPSK) or Gaussian minimum shift keying (GMSK) [9]. Additionally, power imbalance between the in-phase and in-quadrature components also yields an improper signal. A substantial body of work has focused on the design of widely linear receivers to exploit the impropriety of a received improper signal (we refer the interested reader to [10,11,12,13] and the references therein). While these works devise techniques to deal with signals whose impropriety was not intentionally introduced, recent works consider the deliberate transmission of improper signals as a novel way to handle interference in a multiuser channel.

Cadambe et al. showed the benefits of improper signaling for the first time in their pioneering work [2]. They considered a single-antenna three-user interference channel with constant channel extensions and showed that the number of degrees of freedom (DoF), which is the number of interference-free signals that can be transmitted, was larger when users transmit improper signals as opposed to proper ones. Similar DoF results were derived for the four-user interference channel in [14]. After that, a number of new works has emerged, and the payoffs of improper signaling have been revealed for different multiuser scenarios such as the interference channel [3,4,5,15], Z-interference channel [6,16,17,18], broadcast channels with linear precoding [19,20], underlay and overlay cognitive radio networks [7,8,21] and relay channels [22,23]. More recently, in [24], the authors showed the advantages of improper signaling in a multiuser scenario where a point-to-point link coexists with a multiple access channel. Even though these works present new and intriguing results that pave the way for more efficient interference management schemes, the vast majority of them assume Gaussian codebooks (the only exception is [15], where the symbol error rate is minimized for arbitrary digital modulation schemes) and, hence, can never be realized in practice since practical codes use non-Gaussian finite signaling constellations.

It is well known that the penalty in signal-to-noise ratio (snr) paid by constraining the complex symbols to a uniform *M*-ary quadrature amplitude modulation (QAM) constellation is given by the shaping loss, which for large *M* is 1.53 dB in the proper case [25]. When dealing with proper signals, this shaping loss is due to enclosing the uniformly-transmitted codewords inside a hypercube rather than a hypersphere, which would be the optimal region that minimizes the transmitted power.

When using non-Gaussian improper signals, different questions arise: What is the optimal way to generate an improper constellation with a given circularity coefficient (i.e., degree of impropriety)? In coded communication systems, what is the reduction in rate compared to the improper Gaussian capacity when the coded channel symbols are constrained to be in a given discrete improper constellation? Is this rate reduction due only to a shaping loss? Should the channel encoder be modified for discrete improper constellations? In this paper, we address these questions and consider the design of improper discrete constellations for the additive white Gaussian noise channel (AWGN). We focus on coded communication systems, and therefore, our aim is the design of discrete improper constellations for the coded bits to approach the improper Gaussian capacity (i.e., maximize the throughput). Consequently, mutual information is the figure of merit to be optimized. The design of practical capacity-achieving encoders-decoders will be considered in a future work. Therefore, the main contribution of this paper is to propose a class of improper constellations and to show that, in terms of mutual information, this family of constellations behaves very similarly to the standard family of *M*-QAM proper constellations.

Starting with a proper *M*-QAM constellation, we analyze the design of a widely linear transformation that generates a family of improper *M*-QAM constellations with a prescribed circularity coefficient κ. The only free parameter of these improper *M*-ary constellations is a phase ϕ∈[0,π/2]. For uncoded communication systems, this phase should be optimized to maximize the minimum Euclidean distance between points of the improper constellation, so that the bit error rate (BER) is minimized. Note that for a given *M* (i.e., for a given spectral efficiency), maximizing $d_{min}$ is equivalent, at high snr, to maximizing the mutual information (MI) between the transmit and the received signals [26]. On the other hand, for coded improper communication systems with a circularity coefficient κ, one should find the ϕ that maximizes, for each snr and *M*, the envelope of the resulting family of mutual information values of these *M*-ary improper constellations. In this way, the rate gap compared to the improper Shannon capacity is minimized.

One of the main contributions of this paper is to show that except for a shaping loss of 1.53 dB encountered at high snr, there is no rate loss with respect to the improper Gaussian Shannon capacity when using this family of constellations. We conclude that the proposed family of improper constellations behaves much the same as the standard family of proper *M*-QAM constellations. Finally, note that as in the case of proper QAM constellations, the shaping loss can be reduced by designing codes that result in a Gaussian-like rather than uniform distribution of the coded symbols.

The rest of the paper is organized as follows. Section 2 provides preliminaries about improper random variables and introduces the considered model. The design of improper constellations is formulated in Section 3, and its information-theoretic analysis is done in Section 4. Section 5 provides some numerical examples. Finally, concluding remarks are presented in Section 6.

## 2. Background and System Model

### 2.1. Notation

Scalar random variables are denoted by uppercase letters *X*. Vectors and matrices are denoted by lowercase boldface letters, x, and uppercase boldface letters, X, respectively. The determinant and trace of a matrix A will be denoted as det(A) and tr(A). I is the identity matrix. Probability density functions are denoted by $p_{Y}\left(y\right)$. The superscript ${\left(\cdot \right)}^{*}$ denotes complex conjugate, and ${\left(\cdot \right)}^{T}$ denotes transpose. E[·] denotes mathematical expectation, and h(Y) denotes the differential entropy of a continuous random variable. x∼CN(0,R) indicates that x is a complex circular Gaussian random vector of zero mean and covariance R, while $x∼\mathrm{CN}(0,R,\tilde{R})$ indicates that x is a complex non-circular Gaussian random vector of zero mean, covariance R and complementary covariance $\tilde{R}$.

### 2.2. Improper Random Variables

This section introduces some preliminaries about improper random variables that will be used throughout the paper. The interested reader is referred to [9] for a comprehensive treatment of the topic.

**Definition** **1** ([9])**.** *A complex-valued random variable X is said to be circular (or circularly symmetric) if $e^{j\theta }X$ has the same probability distribution as X for all real θ.*

**Definition** **2** ([27])**.** *A complex-valued random variable X is said to be proper if X is uncorrelated with its complex conjugate, i.e., $E[{\left(X-E\left[X\right]\right)}^{2}]=0$.*

A circular *X* is proper, but the converse is not necessarily true. However, for complex Gaussian random variables, propriety implies circularity, so both terms can be used interchangeably. The next definitions characterize an improper random variable.

**Definition** **3** ([9])**.** *The complementary variance of a zero-mean complex random variable X is defined as ${\tilde{\sigma }}_{X}^{2}=E\left[X^{2}\right]$.*

Furthermore, $\sigma_{X}^{2}$ and ${\tilde{\sigma }}_{X}^{2}$ are a valid pair of variance and complementary variance if and only if $\sigma_{X}^{2}\geq 0$ and $|{\tilde{\sigma }}_{X}^{2}|\leq \sigma_{X}^{2}$.

**Definition** **4** ([9])**.** *The circularity coefficient of a complex random variable X, which measures the degree of impropriety, is defined as:*

(1) $$\kappa =\frac{\left|{\tilde{\sigma }}_{X}^{2}\right|}{\sigma_{X}^{2}}.$$

The circularity coefficient satisfies 0≤κ≤1. If κ=0, then *X* is proper, otherwise improper. If κ=1, we call *X* maximally improper.

There are two models that are typically used to express an improper complex random variable $X=X_{I}+jX_{Q}$: the augmented complex model and the real composite model. The former defines the augmented random variable $x={\left[\begin{array}{cc} X & X^{*} \end{array}\right]}^{T}$. This way, its covariance matrix (called the augmented covariance matrix) contains all the second-order information of *X*, i.e.,

(2) $$R_{X}=\left[\begin{array}{cc} \sigma_{X}^{2} & {\tilde{\sigma }}_{X}^{2}\\ {\left({\tilde{\sigma }}_{X}^{2}\right)}^{*} & \sigma_{X}^{2} \end{array}\right].$$

Alternatively, the real composite model defines the real random vector $\tilde{x}={\left[\begin{array}{cc} X_{I} & X_{Q} \end{array}\right]}^{T}$, and its covariance matrix also contains the complete second-order information of *X*. We now present some well-known results about the differential entropy of a complex Gaussian random variable.

**Theorem** **1** ([9])**.** *The differential entropy of an improper complex Gaussian random variable X is:*

(3) $$h\left(X\right)=\frac{1}{2}\log\left[{\left(\pi e\right)}^{2}\det\left(R_{X}\right)\right]=\log\left(\pi e\sigma_{X}^{2}\right)+\frac{1}{2}\log\left(1-\kappa^{2}\right).$$

Taking κ=0 in the above expression yields the entropy of a proper Gaussian random variable [27]. Indeed, it is well known that the entropy of a complex random variable is maximized if it follows a proper complex Gaussian distribution [27].

### 2.3. A Motivating Example

**Figure 1:**
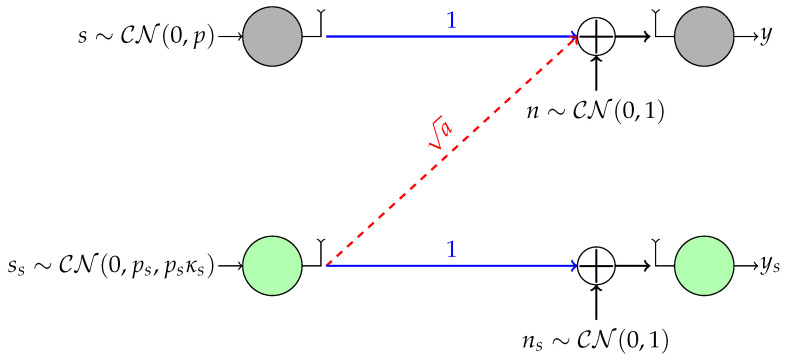
Underlay cognitive radio scenario with a secondary user that may transmit improper Gaussian signals.

In this subsection, we present a motivating example for the study conducted in this paper. Let us consider an underlay cognitive radio (UCR) system transmitting Gaussian codewords in which one single-antenna secondary user (SU) wishes to access the channel in the presence of a single-antenna primary user (PU) as shown in Figure 1, where the top and bottom links represent the PU and the SU, respectively. Without loss of generality, we consider the standard or canonical form of this scenario for which the direct channels are normalized to one, and the noise at both receivers is Gaussian and proper with zero mean and unit variance. This scenario is known in the information theory literature as the Z (or one-sided) interference channel [28].

In the UCR paradigm, the unlicensed SU is allowed to coexist with the PU as long as they ensure a minimum transmission rate ${\bar{R}}_{PU}\geq \xi \log\left(1+p\right)$ for the PU, where ξ∈[0,1] is the loading factor. That is, the transmit power and signaling scheme of the SU must be selected to control the interference level at the primary receiver such that the PU’s rate requirement is guaranteed. Since the PU is typically unaware of the SU, it is also assumed that the PU always transmits proper Gaussian signals, whereas the SU may transmit either proper or improper Gaussian signals depending on which signaling scheme performs better. This problem has been analyzed in [7], where it has been proven that the SU improves its rate by transmitting Gaussian improper signals if and only if (cf. Theorem 1 in [7]):

(4) $$a\geq \beta =1-\frac{p}{{\left(1+p\right)}^{2\xi }-1},$$

where *p* is the power transmitted by the PU. Furthermore, Theorem 1 in [7] also gives us the optimal circularity coefficient $\kappa_{s}$ that must be used by the SU depending on its power budget.

For example, let us suppose that the cross-link channel is a=4; the power budgets for the PU and SU are 100 and 20, respectively; and the loading factor is ξ=0.4. With these parameters β=−1.56 and, since a≥β, improper signaling with $\kappa_{s}=0.96$ (close to maximally improper) improves the rate of the SU. Specifically, the rate achieved by the SU using improper Gaussian codewords is $R_{SU}=3.09$ b/s/Hz, whereas using proper Gaussian codewords, the rate would be only $R_{SU}=2.44$ b/s/Hz.

As we have already argued, practical constraints dictate the use of discrete constellations instead of Gaussian signals. Nevertheless, the previous analysis with Gaussian signals is still valuable as an upper bound in the sense that any finite constellation with the same circularity coefficient will have lower differential entropy than a Gaussian and, consequently, will be less harmful for the PU. Therefore, in our example, for any improper discrete constellation, we might transmit with $\kappa_{s}=0.96$, and the rate requirement of the PU would still be satisfied.

When the PU transmits Gaussian proper signals and the interference at the SU is treated as noise, the SU link is an AWGN channel over which we have to send improper digital signals with a given $\kappa_{s}$. In a more realistic scenario, the PU would also transmit a discrete non-Gaussian constellation. In this case, the interference power tolerated by the PU could potentially vary with respect to Gaussian signaling, and the SU would receive non-Gaussian interference, as well. While all Gaussian signals with a given circularity coefficient are identical in terms of performance, this is not the case with our family of discrete constellations. As we will see shortly, with discrete improper constellations, we have one degree of freedom to optimize the SU performance. In this work, we study a family of improper constellations and design its parameters to maximize the mutual information over the SU link modeled as an AWGN channel. Obviously, if we maximize the mutual information, the SU will cause more harm to the PU, but still, this harm is upper bounded by the harm caused by a Gaussian signal with the same $\kappa_{s}$, so the PU will sustain its rate.

### 2.4. Capacity Results for Gaussian Improper Signals

We consider a complex AWGN channel:

(5) $$Y=g\sqrt{\mathrm{snr}}\;V+N,$$

where N∼CN(0,1) is circular, *V* is the transmitted signal and the channel is a deterministic and known constant, which we take as g=1 without loss of generality.

The capacity (in bits per channel use) of this channel is [1]:

(6) $$C=\underset{p_{V}\left(v\right)}{\sup}I\left(Y;V\right)=\underset{p_{V}\left(v\right)}{\sup}h\left(Y\right)-h\left(Y|V\right)=\underset{p_{V}\left(v\right)}{\sup}h\left(Y\right)-\log\left(\pi e\right),$$

where the supremum is taken over all possible input distributions of *V* with $E[|V{|}^{2}]=1$, and the logarithms have base two. It is well known that the supremum is achieved when V∼CN(0,1) [27].

In this paper, we consider the case where the input signal is unit-power improper with circularity coefficient κ; then, the channel output is also improper Gaussian with variance $E[|Y{|}^{2}]=1+\mathrm{snr}$ and circularity coefficient $\kappa_{y}=\kappa \mathrm{snr}/\left(1+\mathrm{snr}\right)$. By Theorem 1, the differential entropy of *Y* is:

(7) $$h\left(Y\right)=\log\left(\pi e\left(1+\mathrm{snr}\right)\right)+\frac{1}{2}\log\left(1-\kappa_{y}^{2}\right),$$

and the capacity is:

(8) $$C\left(\mathrm{snr},\kappa \right)=\log\left(1+\mathrm{snr}\right)+\frac{1}{2}\log\left(1-\frac{\kappa^{2}}{{\left(1+{\mathrm{snr}}^{-1}\right)}^{2}}\right)\;\;\mathrm{bits}\;\mathrm{per}\;\mathrm{complex}\;\mathrm{dimension}.$$

Observe that for 0<κ≤1, the second term in (8) is always negative, and therefore, there is a capacity loss with respect to proper Gaussian signaling. In Appendix A, we present a heuristic derivation of this formula based on a packing argument similar to [29].

**Remark** **1.** *As snr→∞, the term:*

$$\frac{1}{2}\log\left(1-\frac{\kappa^{2}}{{\left(1+{\mathrm{snr}}^{-1}\right)}^{2}}\right)$$

*in (8) accounts for the loss in capacity due to enclosing the codewords in a multidimensional ellipsoid rather than in a hypersphere (as proper Gaussian signaling does) [30].*

## 3. A Family of Improper Constellations Generated by a Widely Linear Transformation

In this section, we propose a family of improper discrete constellations that are generated by a widely linear transformation of a standard *M*-QAM constellation, where $M=4s^{2}$ with *s* a positive integer. To this end, let $X=X_{I}+jX_{Q}$ be a symbol in a proper *M*-QAM constellation of unit power ($E[|X{|}^{2}]=1$). Then, we propose to generate the corresponding improper symbol as:

(9) $$V=V_{I}+jV_{Q}=h_{1}X+h_{2}X^{*},$$

where $h_{1}=h_{1I}+jh_{1Q}$ and $h_{2}=h_{2I}+jh_{2Q}$ are complex scalars such that $|h_{1}{|}^{2}+{\left|h_{2}\right|}^{2}=1$ (to maintain unit transmit power). Alternatively, the widely linear precoding operation (9) can be expressed in terms of real variables as:

(10) $$\left[\begin{array}{c} V_{I}\\ V_{Q} \end{array}\right]=\left[\begin{array}{cc} h_{1I}+h_{2I} & h_{2Q}-h_{1Q}\\ h_{1Q}+h_{2Q} & h_{1I}-h_{2I} \end{array}\right]\left[\begin{array}{c} X_{I}\\ X_{Q} \end{array}\right].$$

Under the constraint $|h_{1}{|}^{2}+{\left|h_{2}\right|}^{2}=1$, the circularity coefficient of the generated symbol is:

(11) $$\kappa =\frac{\left|E[V^{2}]\right|}{E[|V{|}^{2}]}=2|h_{1}\left|\right|h_{2}|.$$

The question that now arises is: given κ, what is the simplest parametrization of this family of improper constellations in terms of the complex variables $h_{1}$ and $h_{2}$? All values of $h_{1}$ and $h_{2}$ producing a unit variance improper *V* with a given κ must satisfy:

(12) $$\begin{array}{cc} |h_{1}{|}^{2}{\left|h_{2}\right|}^{2} & =\frac{\kappa^{2}}{4},\\ |h_{1}{|}^{2}+{\left|h_{2}\right|}^{2} & =1. \end{array}$$

The nonlinear system of equations in (12) has a unique solution for the squared modulus $|h_{1}{|}^{2}$ and $|h_{2}{|}^{2}$, which can be written in terms of the variable $\alpha =\sqrt{1-\kappa^{2}}$ as:

(13) $$\begin{array}{cc} |h_{1}{|}^{2} & =\frac{1}{2}\left(1+\alpha \right),\\ |h_{2}{|}^{2} & =\frac{1}{2}\left(1-\alpha \right). \end{array}$$

Since for the AWGN channel considered in (5), the noise is Gaussian and proper (circular), it is clear that the mutual information does not change when the constellation is rotated by an arbitrary angle. This means that we can always rotate the constellation *V* such that $h_{1}$ is a real and positive parameter, whereas $h_{2}$ is a complex value. With this restriction, $h_{1}$ and $h_{2}$ can be expressed as:

(14) $$\begin{array}{cc} h_{1} & =\sqrt{\frac{1}{2}\left(1+\alpha \right)}, \end{array}$$

(15) $$\begin{array}{cc} h_{2} & =\sqrt{\frac{1}{2}\left(1-\alpha \right)}e^{j\phi }. \end{array}$$

Furthermore, the original proper *M*-QAM constellation *X* has a rotational symmetry of ±nπ/2 for any integer *n*, which means that ϕ in (15) can be restricted to the interval ϕ∈[0,π/2]. Finally, the proposed family of improper constellations with circularity coefficient κ can be parameterized as:

(16) $$V=\sqrt{\frac{1}{2}\left(1+\alpha \right)}X+\sqrt{\frac{1}{2}\left(1-\alpha \right)}e^{j\phi }X^{*},$$

where $\alpha =\sqrt{\left(1-\kappa^{2}\right)}$, 0≤κ≤1 and ϕ∈[0,π/2]. In what follows, to stress the dependency of the improper constellation on κ, ϕ and *M*, we will denote this family of constellations as A(ϕ,κ,M).

In terms of real variables, the transformation in (16) can be written as:

(17) $$\left[\begin{array}{c} V_{I}\\ V_{Q} \end{array}\right]=\underset{H(\phi ,\kappa )}{\underset{︸}{\left[\begin{array}{cc} \sqrt{0.5(1+\alpha )}+\sqrt{0.5(1-\alpha )}\cos\left(\phi \right) & \sqrt{0.5(1-\alpha )}\sin\left(\phi \right)\\ \sqrt{0.5(1-\alpha )}\sin\left(\phi \right) & \sqrt{0.5(1+\alpha )}-\sqrt{0.5(1-\alpha )}\cos\left(\phi \right) \end{array}\right]}}\left[\begin{array}{c} X_{I}\\ X_{Q} \end{array}\right],$$

where H(ϕ,κ) denotes the 2×2 transformation matrix.

**Figure 2:**
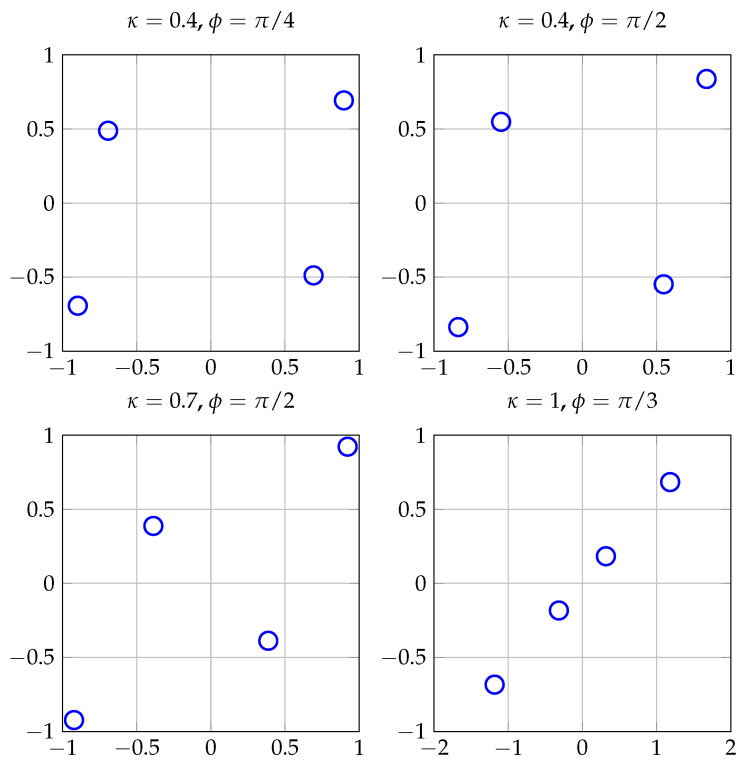
Improper constellations generated according to (16) for different values of κ and ϕ when *X* is proper Quadrature Phase Shift Keying (QPSK).

**Figure 3:**
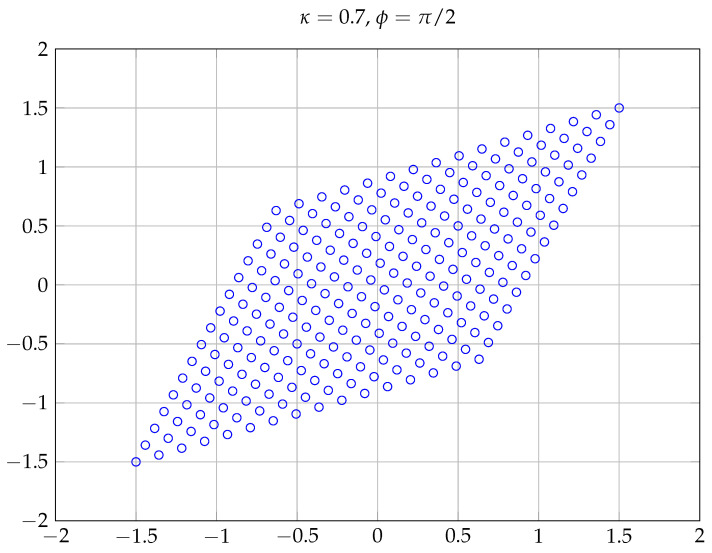
Improper constellation generated according to (16) for κ=0.7 and ϕ=π/2 when *X* is proper 256-QAM (Quadrature Amplitude Modulation).

Observe that by setting ϕ=0 in (16), one obtains a rather naive rectangular QAM improper constellation, where its real and imaginary components are independent and have a power imbalance determined by the required circularity coefficient κ. For any other ϕ≠0 the constellation points are enclosed in a non-rectangular parallelogram. Figure 2 shows four improper constellations generated according to (16) for different values of κ and ϕ when *X* is proper QPSK (M=4). For κ=1 (maximally improper signal), in the right bottom panel, the points of the constellation lie on a line. Similarly, Figure 3 shows the constellation A(π/2,0.7,256), where the shape of the constellation, a parallelogram, can be more clearly observed.

We now provide an alternative geometric interpretation of the proposed improper constellations A(ϕ,κ,M). Consider the 2D integer lattice $Z^{2}\subset R^{2}$, consisting of all two-dimensional vectors $z={\left(a,b\right)}^{T}$ with *a* and *b* integers. Then, the *M*-QAM constellation $\frac{1}{2}{\left[\pm 1,\pm 3,\ldots ,\pm \left(\sqrt{M}-1\right)\right]}^{2}$ can be obtained as the intersection of the translated lattice $Z^{2}+{\left(\frac{1}{2},\frac{1}{2}\right)}^{T}$ and the square region $R={\left[-\frac{\sqrt{M}}{2},\frac{\sqrt{M}}{2}\right]}^{2}\subset R^{2}$.

If we apply the linear transformation H(ϕ,κ) to the lattice $Z^{2}+{\left(\frac{1}{2},\frac{1}{2}\right)}^{T}$, we obtain the new translated lattice $\Lambda \left(\phi ,\kappa \right)+H\left(\phi ,\kappa \right){\left(\frac{1}{2},\frac{1}{2}\right)}^{T}$, where:

(18) $$\Lambda \left(\phi ,\kappa \right)=\{\lambda \in R^{2}:\lambda =H\left(\phi ,\kappa \right)z,\;\forall z\in Z^{2}\}.$$

Reasoning as before, the unnormalized family of improper constellations A(ϕ,κ,M) can be obtained as the intersection of the translated lattice $\Lambda \left(\phi ,\kappa \right)+H\left(\phi ,\kappa \right){\left(\frac{1}{2},\frac{1}{2}\right)}^{T}$ and the parallelogram region $P\subset R^{2}$ that results from the H(ϕ,κ)-transformation of a centered square with side length $\sqrt{M}$, that is,

(19) $$A\left(\phi ,\kappa ,M\right)=\left\{\Lambda \left(\phi ,\kappa \right)+H\left(\phi ,\kappa \right){\left(\frac{1}{2},\frac{1}{2}\right)}^{T}\right\}\cap P.$$

Note that by doing this, only *M* points of the lattice $\Lambda \left(\phi ,\kappa \right)+H\left(\phi ,\kappa \right){\left(\frac{1}{2},\frac{1}{2}\right)}^{T}$ are enclosed, and their average energy is (M−1)/6. Since translation of a lattice does not affect its minimum distance between signal points, $d_{min}$, we conclude that the minimum distance of the unnormalized family of constellations A(ϕ,κ,M) (19) is given by the minimum distance of the lattice Λ(ϕ,κ). For a given κ, the optimal ϕ that maximizes $d_{min}\left(\Lambda \left(\phi ,\kappa \right)\right)$ can be obtained as:

(20) $$\phi^{*}\left(\kappa \right)=\arg\max_{\phi \in [0,\pi /2]}d_{min}\left(\Lambda \left(\phi ,\kappa \right)\right).$$

This problem will be solved in Section 4. Taking into account that $\phi^{*}\left(\kappa \right)$ does not depend on the scaling of the lattice Λ(ϕ,κ), we arrive at the following proposition:

**Proposition** **1.** The angle $\phi^{*}$ that maximizes the minimum distance of the family constellations μ×A(ϕ,κ,M), where μ is an arbitrary normalization factor, only depends on the circularity coefficient κ and is given by (20), where Λ(ϕ,κ) in (18) is the lattice that results from the H(ϕ,κ)-transformation of the integer lattice $Z^{2}$.

## 4. Information-Theoretic Analysis

### 4.1. Introduction

To assess the performance of the proposed family of improper constellations A(ϕ,κ,M) in coded communication systems, one has to check whether the maximum reliable communication rates achievable by channel encoders designed with coded symbols in these constellations are close to the rates dictated by the improper Gaussian capacity (8). To that end, let us define the mutual information envelope, denoted by $I^{*}\left(\mathrm{snr},\kappa \right)$, as follows. Given κ and *M*, we obtain for each snr the optimal phase $\hat{\phi }\left(\kappa ,\mathrm{snr},M\right)$ that maximizes the mutual information I(Y,V). By repeating this optimization process for each *M*, a family of mutual information curves is obtained. $I^{*}\left(\mathrm{snr},\kappa \right)$ is now obtained as the envelope of these curves, that is,

$$I^{*}\left(\mathrm{snr},\kappa \right)=\max_{M}\left\{I\left(Y;V\right):V\in A\left(\hat{\phi }\left(\kappa ,\mathrm{snr},M\right),\kappa ,M\right)\right\}.$$

The closer $I^{*}\left(\mathrm{snr},\kappa \right)$ is to the improper Gaussian capacity C(κ,snr), the better the proposed family of constellations will be. A way of quantifying performance is to obtain the signal-to-noise ratio gap, Δ(snr,κ):

$$C\left(\kappa ,\mathrm{snr}\right)=I^{*}\left(\mathrm{snr}+\Delta \left(\mathrm{snr},\kappa \right),\kappa \right).$$

Although the analytical derivation of Δ(snr,κ) looks intractable, useful insights into the behavior of Δ(snr,κ) versus snr can be gained by analyzing in the high- and low-snr regimes the family of mutual information values, $\left\{I\left(Y;V\right):V\in A\left(\hat{\phi }\left(\kappa ,\mathrm{snr},M\right),\kappa ,M\right),\;\mathrm{for}\;\mathrm{all}\;M\;\mathrm{and}\;\mathrm{snr}\;\mathrm{values}\right\}$. This is studied in the following sections.

Section 4.2 shows that at low snr, the behavior of C(κ,snr) and $I^{*}\left(\mathrm{snr},\kappa \right)$ is the same up to the second order in snr. In Section 4.3, we show that at high snr, the optimal phase $\hat{\phi }\left(\kappa ,\mathrm{snr},M\right)$ that maximizes the mutual information I(Y;V) with V∈A(ϕ,κ,M) does not depend on *M* and is given by $\phi^{*}\left(\kappa \right)$, i.e., the phase that maximizes the minimum distance of the lattice Λ(ϕ,κ) (refer to Proposition 1). Observe that maximizing the mutual information in this context is equivalent to minimizing the BER for uncoded systems.

In Section 4.4, we compute the shaping loss of the proposed family of constellations A(ϕ,κ,M) with respect to improper Gaussian signaling. It is shown that this loss is 1.53 dB, for all ϕ and κ. Although this result can easily be checked for rectangular QAM improper constellations (when ϕ=0), it has to be proven for any other value of ϕ∈(0,π/2], which generates non-rectangular constellations.

Section 5 shows by computer simulation that the gap, Δ(snr,κ), increases monotonically with the snr up to a saturation value of 1.53 dB. Since at low snr, the value of Δ(snr,κ) is negligible, we conclude that the only loss in rate is due to shaping. In this sense, the proposed family of constellations can be viewed as the improper counterpart of the standard proper *M*-QAM constellations.

### 4.2. Asymptotic Results at Low SNR

The following theorem characterizes the mutual information at low snr up to the second order.

**Theorem** **2.** *Let V be a discrete improper random variable with circularity coefficient 0≤κ≤1 taking values in A(ϕ,κ,M) and constructed as in (16) with X being a square proper M-QAM constellation. Then, the mutual information between Y and V in (5) admits the following second-order expansion around snr=0:*

(21) $$I\left(\sqrt{\mathrm{snr}}\;V+N;V\right)=\mathrm{snr}-\frac{1}{2}\left(1+\kappa^{2}\right){\mathrm{snr}}^{2}+O\left({\mathrm{snr}}^{3}\right).$$

**Proof.**  From Expression (17), the random variable $V=V_{I}+jV_{Q}$ can be expressed in terms of real variables as:

(22) $$\underset{v}{\underset{︸}{\left[\begin{array}{c} V_{I}\\ V_{Q} \end{array}\right]}}=\underset{H(\phi ,\kappa )}{\underset{︸}{\left[\begin{array}{cc} \sqrt{0.5(1+\alpha )}+\sqrt{0.5(1-\alpha )}\cos\left(\phi \right) & \sqrt{0.5(1-\alpha )}\sin\left(\phi \right)\\ \sqrt{0.5(1-\alpha )}\sin\left(\phi \right) & \sqrt{0.5(1+\alpha )}-\sqrt{0.5(1-\alpha )}\cos\left(\phi \right) \end{array}\right]}}\underset{x}{\underset{︸}{\left[\begin{array}{c} X_{I}\\ X_{Q} \end{array}\right]}},$$

where $\alpha =\sqrt{1-\kappa^{2}}$ and $X_{I}+jX_{Q}$ is a proper uniformly-distributed *M*-QAM random variable. Then, the complex-valued AWGN channel reduces to the real-valued vector channel:

(23) y=H(ϕ,κ)x+n,

where $y={\left(Y_{I},Y_{Q}\right)}^{T}$ and $n={\left(N_{I},N_{Q}\right)}^{T}$, with $N_{I}$ and $N_{Q}$ being independent real Gaussian random variables with zero mean and variance $\frac{1}{2}$.

We exploit the known relationship between mmse and mutual information (MI) [31], namely,

(24) $$\frac{d}{d\;\mathrm{snr}}I\left(\sqrt{\mathrm{snr}}\right)=\mathrm{mmse}\left(\sqrt{\mathrm{snr}}\right),$$

and use a result in [31] showing that the mmse of a real-valued vector channel can be expanded around snr=0 up to the first order as (notice that the factor 1/2 in the expression is because, according to our formulation, $E\left[xx^{T}\right]=\frac{1}{2}I$):

(25) $$\mathrm{mmse}\left(\mathrm{snr},\phi ,\kappa \right)=\frac{1}{2}\mathrm{tr}\left(H\left(\phi ,\kappa \right)H{\left(\phi ,\kappa \right)}^{T}\right)-\frac{1}{2}\mathrm{snr}\cdot \mathrm{tr}\left({\left(H\left(\phi ,\kappa \right)H{\left(\phi ,\kappa \right)}^{T}\right)}^{2}\right)+O\left({\mathrm{snr}}^{2}\right).$$

Since the matrix H(ϕ,κ) is real and symmetric, it is always diagonalizable as:

$$H\left(\phi ,\kappa \right)=Q\Lambda Q^{T},$$

where Q is an orthogonal matrix (${\mathrm{QQ}}^{T}=Q^{T}Q=I$) and:

$$\Lambda =\mathrm{diag}\{\lambda_{1}\left(\phi ,\kappa \right),\lambda_{2}\left(\phi ,\kappa \right)\},$$

with $\lambda_{j}\left(\phi ,\kappa \right)$, j=1,2, being its real eigenvalues. It is easy to check that:

$$\lambda_{1}\left(\phi ,\kappa \right)=\sqrt{0.5(1+\alpha )}-\sqrt{0.5(1-\alpha )},$$

$$\lambda_{2}\left(\phi ,\kappa \right)=\sqrt{0.5(1+\alpha )}+\sqrt{0.5(1-\alpha )}.$$

Note that these eigenvalues do not depend on ϕ. By the properties of the trace operator $\mathrm{tr}\left(H\left(\phi ,\kappa \right)H{\left(\phi ,\kappa \right)}^{T}\right)=\lambda_{1}^{2}\left(\phi ,\kappa \right)+\lambda_{2}^{2}\left(\phi ,\kappa \right)=2$ and $\mathrm{tr}\left({\left(H\left(\phi ,\kappa \right)H{\left(\phi ,\kappa \right)}^{T}\right)}^{2}\right)=\lambda_{1}^{4}\left(\phi ,\kappa \right)+\lambda_{2}^{4}\left(\phi ,\kappa \right)=2\left(1+\kappa^{2}\right)$.

Therefore, Expression (25) reduces to:

(26) $$\mathrm{mmse}\left(\mathrm{snr},\kappa \right)=1-\left(1+\kappa^{2}\right)\mathrm{snr}+O\left({\mathrm{snr}}^{2}\right),$$

and using the relationship (24), we obtain:

(27) $$I\left(\sqrt{\mathrm{snr}}\;V+N;V\right)=\mathrm{snr}-\frac{1}{2}\left(1+\kappa^{2}\right){\mathrm{snr}}^{2}+O\left({\mathrm{snr}}^{3}\right),$$

as we wanted to prove. ☐

**Remark** **2.** Theorem 2 is an extension to improper signaling (and up to the second-order term) of the well-known result that states that at low snr, the proper Gaussian capacity behaves linearly with snr.

**Remark** **3.** Equation (21) shows that at low snr, the behavior of the proposed family of improper constellations A(ϕ,κ,M) only depends on the circularity coefficient κ, but neither on the phase ϕ, nor on the cardinality M of V.

A more elaborate proof derived directly on the complex field gives us the MI third-order expansion around snr=0. This is stated in the next theorem, which is proven in Appendix B.

**Theorem** **3.** *Let V be a discrete improper random variable with circularity coefficient 0≤κ≤1 taking values in A(ϕ,κ,M) and constructed as in (16) with X being a square proper M-QAM constellation. Then, the mutual information between Y and V in (5) admits the following third-order expansion around snr=0:*

(28) $$I\left(\sqrt{\mathrm{snr}}\;V+N;V\right)=\mathrm{snr}-\frac{1}{2}\left(1+\kappa^{2}\right){\mathrm{snr}}^{2}+\frac{1}{3}\left(1-\kappa^{2}\right){\mathrm{snr}}^{3}+O\left({\mathrm{snr}}^{4}\right).$$

**Proof.**  See Appendix B. ☐

**Remark** **4.** *The asymptotic expansion of the capacity (8) of the AWGN channel when transmitting improper Gaussian codewords is:*

(29) $$C\left(\mathrm{snr},\kappa \right)=\mathrm{snr}-\frac{1}{2}\left(1+\kappa^{2}\right){\mathrm{snr}}^{2}+\frac{1}{3}\left(1+3\kappa^{2}\right){\mathrm{snr}}^{3}+O\left({\mathrm{snr}}^{4}\right).$$

Comparing Equations (28) and (29), we see that at low snr, the behavior of the mutual information for the discrete improper constellation V and the improper Gaussian capacity is the same up to the second-order term.

### 4.3. Asymptotic Results at High SNR

The optimum $\hat{\phi }$ that maximizes $I(\sqrt{\mathrm{snr}}\;V+N;V)$ depends in general on *M*, κ and the snr. The analytical derivation of $\hat{\phi }\left(\mathrm{snr},\kappa ,M\right)$ for all snr appears to be intractable in general. However, at high snr, the optimal phase depends only on κ and is given by the following theorem.

**Theorem** **4.** At high snr, the optimal $\hat{\phi }$ that maximizes $I(\sqrt{\mathrm{snr}}\;V+N;V)$ over $0\leq \phi \leq \frac{\pi }{2}$ is equal to $\phi^{*}\left(\kappa \right)$ in Expression (20) and is given by:

(30) $$\lim_{\mathrm{snr}\to \infty }\hat{\phi }\left(\mathrm{snr},\kappa ,M\right)=\phi^{*}\left(\kappa \right)=\left\{\begin{array}{cc} \pi /2, & 0<\kappa \leq 0.5,\\ \phi^{*}, & 0.5<\kappa \leq 1, \end{array}\right.$$

where $\phi^{*}$ is the solution of the equation 2sin(ϕ)−cos(ϕ)=1/κ.

**Proof.**  In order to proceed, we use the fact [26] that for any discrete constellation, the mutual information, the minimum mean-squared error (mmse) and the symbol error probability all have an asymptotic behavior proportional to $Q\left(\sqrt{\mathrm{snr}}d_{min}/2\right)$, where Q(·) denotes the Gaussian Q-function and $d_{min}$ is the minimum Euclidean distance of the constellation.

In Proposition 1, we showed that the value of $\phi^{*}\left(\kappa \right)$ that maximizes the minimum distance of the family of constellations A(ϕ,κ,M) is given by:

(31) $$\phi^{*}\left(\kappa \right)=\arg\max_{\phi \in [0,\pi /2]}d_{min}\left(\Lambda \left(\phi ,\kappa \right)\right)$$

where Λ(ϕ,κ) is the lattice that results from the H(ϕ,κ)-transformation of the integer lattice $Z^{2}$ (refer to (18)). From (18) and exploiting the fact that 0≤ϕ≤π/2, it can be computed that:

$$d_{min}^{2}=\min\left\{\left(1-\kappa \cos(\phi )\right),2\left(1-\kappa \sin(\phi )\right)\right\},$$

which can be obtained from the real representation given by (17) when the signal points of the original proper constellation belong to a regular square lattice. It is easy to check that $0\leq \kappa \leq 0.5\Rightarrow \left(1-\kappa \cos(\phi )\right)\leq 2\left(1-\kappa \sin(\phi )\right)$. This, in turn, means that the optimal solution of (31) when 0≤κ≤0.5 is $\phi^{*}\left(\kappa \right)=\pi /2$. On the other hand, when κ>0.5, the optimal phase satisfies the nonlinear equation:

(32) $$\left(1-\kappa \cos(\phi )\right)=2\left(1-\kappa \sin(\phi )\right)\Leftrightarrow 2\sin\left(\phi \right)-\cos\left(\phi \right)=\frac{1}{\kappa },$$

which proves the theorem. ☐

**Remark** **5.** For κ=1 (maximally improper, rectilinear, constellations), the optimal phase in Equation (30) is $\phi^{*}\approx 0.9273$. This value results in a rectilinear constellation with equidistant points, which would be equivalent to a rotated M-ary pulse amplitude modulation (PAM). As an example, starting from a standard QPSK constellation and using κ=1 and $\phi^{*}\approx 0.9273$ in (16), we obtain a unit-norm rectilinear constellation with equidistant signal points:

$$V=\left[\begin{array}{cccc} 1.2+0.6i & 0.4+0.2i & -0.4-0.2i & -1.2-0.6i \end{array}\right].$$

### 4.4. Shaping Loss

Next, we compute the shaping loss defined as the ratio of the powers required to achieve the same transmission rate when using symbols that are either uniformly distributed in A(ϕ,κ,M) (by assuming *M* to be an unbounded large number) or Gaussian distributed. The shaping loss in the improper case can also be interpreted as the power penalty due to using codewords uniformly distributed inside a multidimensional parallelogram rather than inside a multidimensional ellipsoid.

**Theorem** **5.** *The shaping loss $\gamma_{s}$ of the proposed family of improper constellations with respect to improper Gaussian signaling is:*

$$\gamma_{s}=\frac{\pi \;e}{6}\;\;\left(1.53\;dB\right)$$

*which is independent of κ and ϕ and coincides with the shaping loss of proper M-QAM constellations [25].*

**Proof.**  The shaping gain can be obtained as follows. Let us consider two memoryless complex sources. The first source generates discrete values $V=V_{I}+jV_{Q}$ uniformly distributed over the constellation A(ϕ,κ,M) where *M* is assumed to be a very large number. Under this assumption, its uniform distribution can be approximated by the probability density function:

$$f_{V}\left(v_{I},v_{Q}\right)=\left\{\begin{array}{cc} \frac{1}{A(P)} & \mathrm{if}\;(v_{I},v_{Q})\in P;\\ 0 & \mathrm{otherwise}, \end{array}\right.$$

where P denotes the parallelogram determined by the boundaries of the constellation A(ϕ,κ,M) and A(P) denotes its area. Based on the Jacobian of the transformation H(ϕ,κ), this area can be computed as:

A(P)=det(H(ϕ,κ))A(R)

where A(R)=1 denotes the area of the square $R={\left[-0.5,0.5\right]}^{2}$. Note that the second-order moment of R is given by $P_{R}=1/6$ and so will be the second-order moment, $P_{V}$, of the parallelogram P, since the transformation H(ϕ,κ) keeps its input-output power invariant. Therefore, A(P) can also be written as:

$$A\left(P\right)=\det\left(H\left(\phi ,\kappa \right)\right)A\left(R\right)=\sqrt{1-\kappa^{2}}\;6\;P_{V}.$$

The second source, $G=G_{I}+jG_{Q}$, generates continuous values in C, with the improper complex Gaussian distribution:

$$f_{G}\left(g_{I},g_{Q}\right)=N\left(0,K_{G}\right),$$

where $K_{G}=\mathrm{diag}\left(\frac{P_{G}}{2}\left(1+\kappa \right),\frac{P_{G}}{2}\left(1-\kappa \right)\right)$ and $P_{G}$ denotes the second-order moment of this Gaussian source.

Note that in order to generate the same number of bits per source symbol (i.e., the same information rate), the entropy of both sources should be the same. Since:

$$h_{V}\left(V_{I},V_{Q}\right)=\int \int_{A(P)}\log A\left(P\right)\frac{1}{A(P)}dv_{I}dv_{Q}=\log A\left(P\right)=\log\left(\sqrt{1-\kappa^{2}}\;6\;P_{V}\right)$$

and:

$$h_{G}\left(G_{I},G_{Q}\right)=\log\left(\left(2\pi e\right)\sqrt{\det(K_{G})}\right)=\log\left(\left(2\pi e\right)\frac{P_{G}}{2}\sqrt{1-\kappa^{2}}\right),$$

we find that $\gamma_{s}$ is:

$$\gamma_{s}=\frac{P_{V}}{P_{G}}=\frac{\pi e}{6},$$

as we wanted to prove. ☐

## 5. Numerical Results

### 5.1. Mutual Information as a Function of ϕ

**Figure 4:**
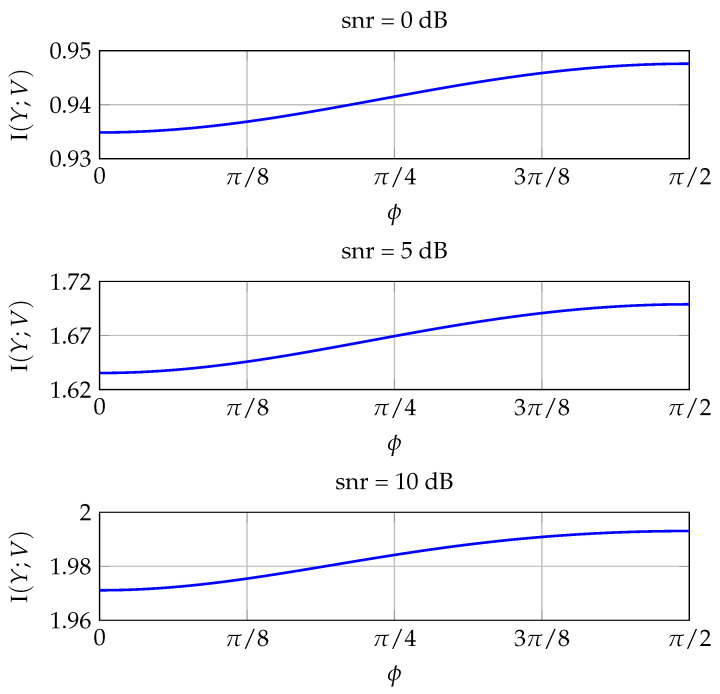
Mutual information of an improper QPSK constellation with κ=0.4 as a function of ϕ, for three different signal-to-noise ratio (snr).

**Figure 5:**
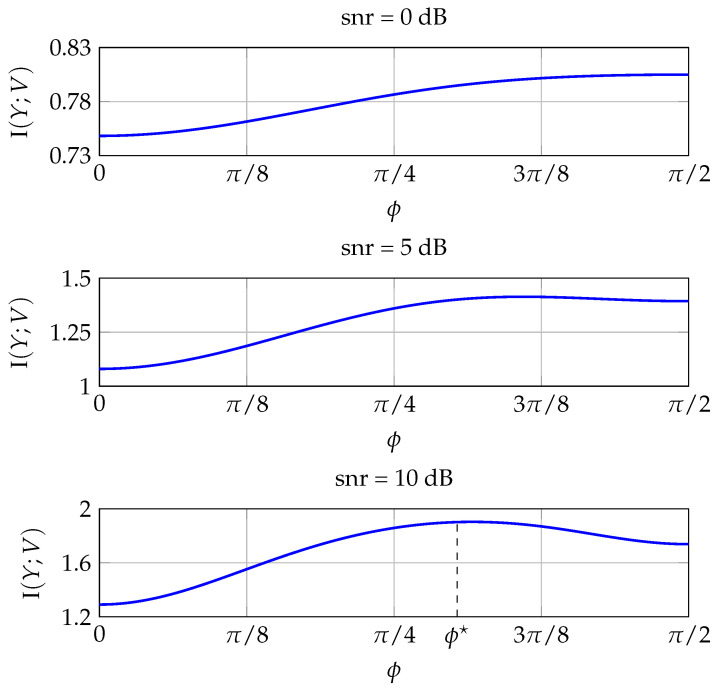
Mutual information of an improper QPSK constellation with κ=0.95 as a function of ϕ, for three different snr. The optimal phase is $\phi^{*}=0.9538$.

In this section, we present some numerical results that validate our findings. First, we compute numerically the MI of improper constellations generated according to (16) and study its behavior with respect to ϕ. Figure 4 plots the MI of an improper QPSK constellation with κ=0.4 for increasing values of the snr. The MI always peaks at ϕ=π/2, which is the optimal value according to Theorem 4. Note the different scale of the y-axis for each subplot. Figure 5 shows the results for an improper QPSK signal with a higher circularity coefficient (κ=0.95). In this case, as the snr increases, the optimal phase decreases until reaching, for sufficiently high snr, the optimal $\phi^{*}$ in Theorem 4. For κ=0.95, the optimal phase shown in Figure 5 is $\phi^{*}=0.9538$.

**Figure 6:**
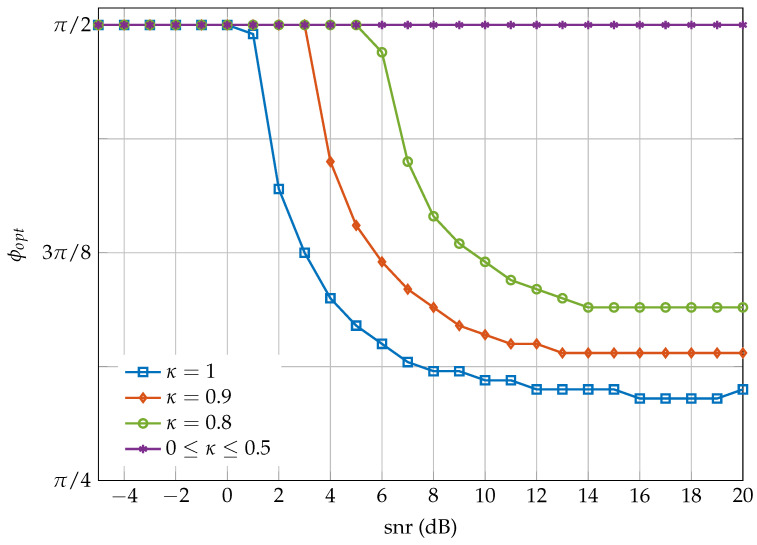
$\phi_{opt}$ versus snr in dBs for different values of κ.

Finally, Figure 6 shows the value of ϕ that maximizes I(Y;V) for different values of κ for an improper QPSK constellation. In the interval 0≤κ≤0.5, the optimum value is π/2 regardless of the snr. When κ > 0.5, π/2 is still the optimum value for the low-snr regime, whereas at high snr the optimum value is $\phi_{opt}=\phi^{*}$ given by Equation (30), which depends only on κ. The curves in Figure 6 for κ > 0.5 are monotonically non-increasing. However, for high values of both κ and snr, the estimated curves present some small fluctuations, which are due to numerical errors in the simulation. These errors can be explained because the mutual information is computed by numerical integration over a finite region for a discrete grid of equally-spaced phases.

### 5.2. Capacity Curves

**Figure 7:**
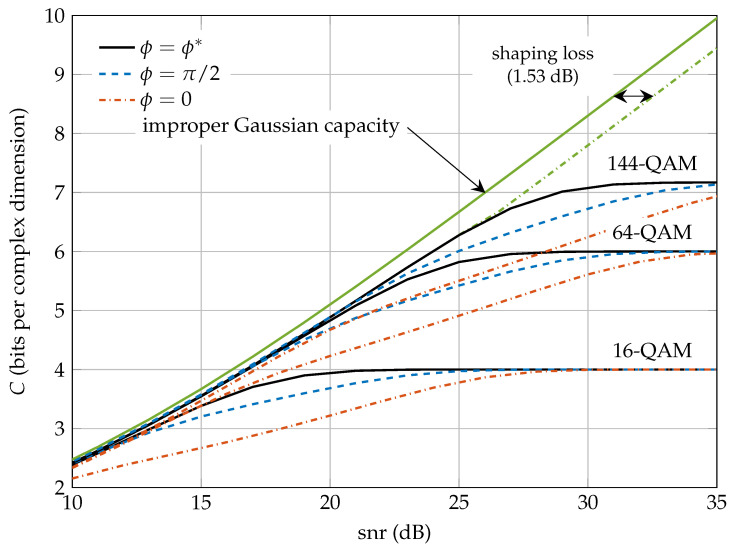
Mutual information vs. snr when κ=0.95 for ϕ=π/2 (blue dashed lines), ϕ=0 (red dashed lines) and $\phi^{*}=0.9538$ (black solid lines) for improper *M*-QAM signals. The capacity of the improper Additive White Gaussian Noise (AWGN) channel using Gaussian codewords and the asymptotic envelope of the curves are also depicted.

Figure 7 plots the mutual information achieved by improper *M*-QAM signals with κ=0.95 when the optimum $\phi^{*}=0.9538$ at high snr is used (black solid lines), in comparison to the case when we use fixed ϕ=π/2 (dashed blue lines) or ϕ=0 (red dashed lines). We consider three increasing values of the constellation size: M= 16, 64 and 144. The constellation with ϕ=0 is a rectangular constellation with uncorrelated real and imaginary parts, but with a power imbalance to achieve the desired circularity coefficient. We observe that this naive family A(ϕ=0,κ,M) of constellations attains lower MI than using the optimum $\phi^{*}=0.9538$ given by Theorem 4. This rectangular constellation is also outperformed by the constellation generated with ϕ=π/2.

The figure also depicts the Gaussian capacity curve with improper signaling and the envelope of the capacity curves with *M*-QAM constellations for *M* sufficiently large. The distance between both curves is the shaping gain, which as proven in Theorem 5 is 1.53 dB. It should be mentioned that, since the shaping loss does not depend on ϕ, the mutual information envelope $I^{*}\left(\mathrm{snr},\kappa \right)$ obtained from the set of constellations with ϕ=0, ϕ=π/2 or with the optimum $\phi^{*}$ are all the same as *M* increases. However, for a given snr and κ, the minimum *M* that makes $I\left(Y;V\right)\approx I^{*}\left(\mathrm{snr},\kappa \right)$, will be smaller when $\phi =\phi^{*}\left(\kappa \right)$ than when ϕ=0. That is, ϕ=0 needs a larger constellation expansion ratio [30] to achieve the same rate.

**Figure 8:**
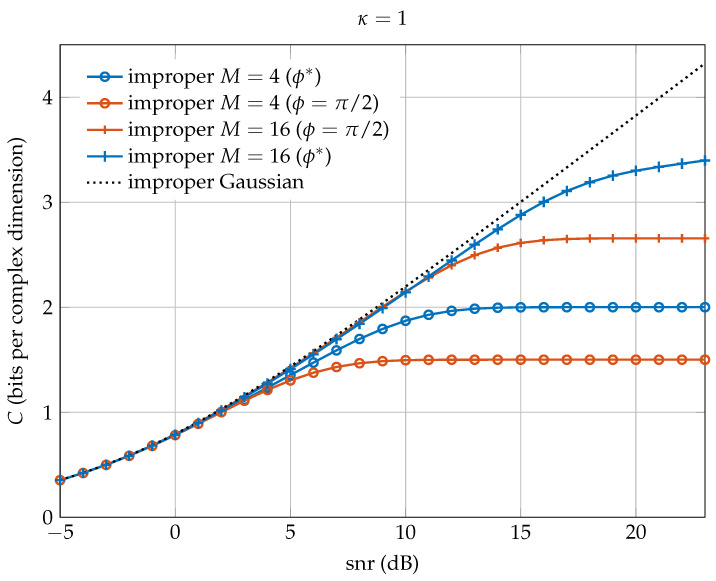
Mutual information vs. snr when κ=1 (maximally improper) for ϕ=π/2 and $\phi^{*}$ for improper constellations with M=4 and M=16 signal points. The capacity of the improper AWGN channel using Gaussian codewords is also depicted.

The situation is different for maximally improper signals (κ=1) (i.e., rectilinear constellations). In this case, using ϕ=π/2 makes some signal points of the original proper constellation collapse into the same symbol, thus losing rate at high snr. This effect becomes evident in Figure 8, which plots the mutual information achieved by the proposed improper constellation with κ=1, and M=4 and M=16 signal points, for ϕ=π/2 and for the optimal $\phi^{*}=0.9273$ at high snr. From a practical point of view, we advocate the use of the optimal $\phi^{*}$ given in (30), which maximizes the minimum distance between constellation points.

**Figure 9:**
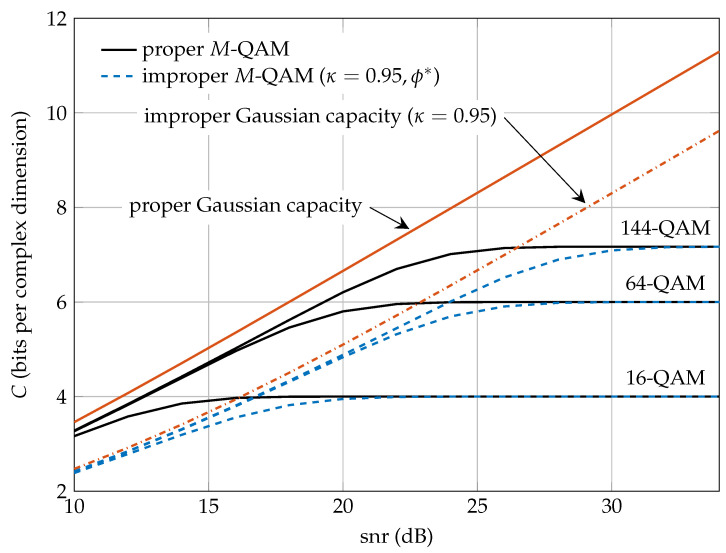
Capacity comparison between proper and improper (with κ = 0.95) *M*-QAM discrete constellations. For the improper *M*-QAM, the optimal $\phi^{*}=0.9538$ at high snr is used. The proper and improper Gaussian capacity is also depicted.

Finally, Figure 9 depicts the capacity curves for proper and improper (with κ = 0.95) *M*-QAM discrete constellations. For the improper *M*-QAM, the optimal $\phi^{*}=0.9538$ at high snr is used. The proper and improper Gaussian capacity is also depicted for comparison.

From these results, the following conclusions are drawn:

1. For low-rate transmissions (M=4 or M=16), there is no rate loss with respect to the improper Gaussian capacity when using coded symbols uniformly drawn from the proposed family of improper constellations. Therefore, like in the proper case, the proposed improper QPSK and 16-QAM constellations are optimal in the wideband regime [32].
2. For high-rate transmissions (M>16), the proposed family of improper constellations incurs a shaping loss of up to 1.53 dB. As in the case of proper *M*-QAM signaling, this snr loss can be reduced by designing codes with some shaping mechanism to make the coded symbols in A(ϕ,κ,M) more Gaussian-like distributed.
3. The optimal $\phi^{*}$, derived at high snr, can be safely used without any observable degradation in Δ(snr,κ), independently of the value of *M* and snr.

Therefore, we arrive at the main conclusion of the paper, namely that the proposed family of improper constellations is a good candidate to be used in communication systems with improper signaling. In fact, it can be viewed as the improper counterpart of the standard proper *M*-QAM constellations.

## 6. Conclusions

In this paper, we have proposed a family of improper constellations constructed by widely linear processing of proper *M*-QAM signals. We have shown that, when the coded channel symbols are uniformly drawn from these constellations, the achievable transmission rates are close to the ones dictated by the improper Gaussian capacity. In particular, except for a shaping loss of 1.53 dB encountered at high snr, there is no rate loss of the proposed improper discrete constellations with respect to the improper Shannon capacity. In practice, we can select the optimal phase at high snr, $\phi^{*}\left(\kappa \right)$, obtained by minimizing the BER of the uncoded system without significant loss in performance at lower snr. Observe that this fact facilitates the practical implementation of these constellations since $\phi^{*}\left(\kappa \right)$ does not depend on the snr and *M*.

These results are obtained by analyzing at the high- and low-snr regimes the family of mutual information, $\left\{I\left(Y;V\right):V\in A\left(\phi^{*}\left(\kappa ,\mathrm{snr},M\right),\kappa ,M\right),\;\mathrm{for}\;\mathrm{all}\;M\;\mathrm{and}\;\mathrm{snr}\;\mathrm{values}\right\}$, and by obtaining the shaping loss computed as the ratio of the normalized second moments of a multidimensional parallelogram and a multidimensional ellipsoid.

We conclude that the proposed family of improper constellations is a good candidate to be used in communication systems when improper signaling is required over the AWGN channel. In fact, it behaves like the standard family of proper *M*-QAM constellations and can be viewed as its improper counterpart. In perspective, these results extend the analysis of improper signaling in interference-limited scenarios to non-Gaussian discrete constellations with equiprobable symbols.

## Abbreviations

The following abbreviations are used in this manuscript:

AWGN	Additive white Gaussian noise
BPSK	Binary phase shift keying
GMSK	Gaussian minimum shift keying
MI	Mutual information
MMSE	Minimum mean-squared error
PAPR	Peak-to-average-power ratio
PU	Primary user
QAM	Quadrature amplitude modulation
QPSK	Quadrature phase shift keying
SNR	Signal-to-noise ratio
SU	Secondary user
UCR	Underlay cognitive radio

## Appendix A. Derivation of the Capacity for Improper Gaussian Signals by an Elliptic Packing Argument

We want to reliably transmit $2^{NR}$ uniformly-distributed messages across an AWGN channel. To that end, we associate with each message $m\in \{1,2,\ldots ,2^{NR}\}$ a complex point x(m) belonging to some *N*-dimensional regular lattice with minimum Euclidean distance $d_{min}$ and enclosed in some region $R\subset C^{N}$. This region is such that the induced empirical distribution on each of the *N* complex components satisfies the improper circularity constraint:

(A1) $$\frac{|E\left[X_{k,I}^{2}\right]-E\left[X_{k,Q}^{2}\right]|}{E\left[X_{k,I}^{2}\right]+E\left[X_{k,Q}^{2}\right]}=\kappa ,\;\forall k=1,2,\ldots ,N,$$

where $X_{k,I}$ and $X_{k,Q}$ denote the real and complex components of symbol *k*. Without any loss of generality, we also assume $E[X_{k,I}X_{k,Q}]=0$

The total average energy of such constellation for large *N* can be approximated as:

$$P_{T}=\frac{1}{2^{nR}}\sum_{k=1}^{2^{nR}}{∥x\left(k\right)∥}^{2}\approx \int_{R}{∥r∥}^{2}\frac{dr}{\mathrm{Vol}(R)},$$

The region R that minimizes $P_{T}$ (i.e., the second moment of R), under the constraint (A1), is the *N*-dimensional ellipsoid:

(A2) $$E_{X}=\left\{r={\left(r_{1,I},r_{1,Q},\ldots ,r_{N,I},r_{N,Q}\right)}^{T}\in R^{2N}:\frac{1}{\sigma_{I}^{2}}∥r_{I}{∥}^{2}+\frac{1}{\sigma_{Q}^{2}}{∥r_{Q}∥}^{2}=2N\left(1+\epsilon \right)\right\}.$$

where $E\left[X_{k,I}^{2}\right]=\sigma_{I}^{2}$, $E\left[X_{k,Q}^{2}\right]=\sigma_{Q}^{2}$ for all k=1,2,…,N and $\sigma_{Q}^{2}=\sigma_{I}^{2}\frac{1-\kappa }{1+\kappa }$.

By the law of large numbers, the *N*-dimensional received complex vector y=x+n will lie, with high probability, within the ellipsoid:

(A3) $$E_{Y}=\left\{r={\left(r_{1,I},r_{1,Q},\ldots ,r_{N,I},r_{N,Q}\right)}^{T}\in R^{2N}:\frac{1}{\sigma_{I}^{2}+N_{0}/2}∥r_{I}{∥}^{2}+\frac{1}{\sigma_{Q}^{2}+N_{0}/2}{∥r_{Q}∥}^{2}=2N\left(1+\epsilon \right)\right\},$$

and hence, without loss of generality, we only need to focus on what happens inside this $E_{Y}$ ellipsoid. On the other hand,

$$\lim_{N\to \infty }\frac{1}{N}\sum {\left|N_{k}\right|}^{2}=N_{0},$$

again by the law of large numbers. That is, the noise realizations of the channel are with high probability, inside a sphere $S_{N}$ of radius $\sqrt{N\;N_{0}}$.

Therefore, when the codeword x(m) is sent, the received vector at the output of the channel will be contained with high probability in the sphere $S_{N}$ centered at x(m). If we assign as the decision region for message *m* the region $D_{m}=x\left(m\right)+S_{N}$, an error will be produced only if the received vector falls outside $D_{m}$, which has low probability. By choosing the other decision regions in the same way, i.e., $D_{l}=x\left(l\right)+S_{N}$, the overall probability of error will be negligible, as long as there is no intersection among the spheres. Therefore, the maximum number of possible messages, i.e., $2^{nC}$ is given by the ratio:

(A4) $$M=\frac{\mathrm{Vol}(E_{Y})}{\mathrm{Vol}(S_{N})}.$$

The volumes of the $E_{Y}$ ellipsoid and the $S_{N}$ sphere are [33]:

(A5) $$\mathrm{Vol}\left(E_{Y}\right)=\frac{{\left(2N\pi \right)}^{N}}{N!}{\left(\sigma_{Q}^{2}\sigma_{I}^{2}\right)}^{\frac{N}{2}}\;\;\;\mathrm{and}\;\;\;\mathrm{Vol}\left(S_{N}\right)=\frac{{\left(2N\pi \right)}^{N}}{N!}{\left(\frac{N_{0}}{2}\right)}^{N},$$

respectively.

Substituting (A5) into (A4) yields:

$$M=\frac{\mathrm{Vol}(E_{Y})}{\mathrm{Vol}(S_{N})}=\frac{{\left(P\left(1+\kappa \right)+N_{0}\right)}^{\frac{N}{2}}{\left(P\left(1-\kappa \right)+N_{0}\right)}^{\frac{N}{2}}}{N_{0}^{N}}=2^{\frac{N}{2}\log\left({\left(1+\mathrm{snr}\right)}^{2}-\kappa^{2}{\mathrm{snr}}^{2}\right)},$$

where $P=\sigma_{I}^{2}+\sigma_{Q}^{2}$ is the average power per complex dimension. Therefore,

$$C=\frac{\log M}{N}=\frac{1}{2}\log\left\{{\left(1+\mathrm{snr}\right)}^{2}-\kappa^{2}{\mathrm{snr}}^{2}\right\}=\log\left(1+\mathrm{snr}\right)+\frac{1}{2}\log\left(1-\frac{\kappa^{2}}{{\left(1+{\mathrm{snr}}^{-1}\right)}^{2}}\right),$$

which is the capacity of the AWGN channel with improper Gaussian codewords in Equation (8).

## Appendix B. Proof of Theorem 3

For the proof, we follow the technique used in [31,34]. Let us denote the standard complex Gaussian density as $\psi \left(y\right)=\frac{1}{\pi }e^{-{\left|y\right|}^{2}}$, and let us define the functions:

(A6) $$\begin{array}{cc} g_{0}\left(y;\mathrm{snr}\right) & =E\left[\psi (y-\sqrt{\mathrm{snr}}V)\right], \end{array}$$

(A7) $$\begin{array}{cc} g_{1}\left(y;\mathrm{snr}\right) & =E\left[V\psi (y-\sqrt{\mathrm{snr}}V)\right], \end{array}$$

where the expectation is taken over *V*. The conditional mean estimate can be expressed as [31]:

(A8) $$E\left[V|Y=y\right]=\frac{g_{1}\left(y;\mathrm{snr}\right)}{g_{0}\left(y;\mathrm{snr}\right)},$$

and (taking into account that $E[|V{|}^{2}]=1$), the mmse can be calculated as [34]:

(A9) $$\mathrm{mmse}\left(\mathrm{snr}\right)=1-\int \frac{|g_{1}{\left(y;\mathrm{snr}\right)|}^{2}}{g_{0}\left(y;\mathrm{snr}\right)}dy,$$

where the integral is calculated over the complex field.

When snr→0, the Gaussian complex density can be expanded as:

(A10) $$\psi \left(y-\sqrt{\mathrm{snr}}V\right)=\psi \left(y\right)\left[1+\left(y^{*}V+yV^{*}\right)\sqrt{\mathrm{snr}}+\frac{1}{2}({\left(y^{*}V\right)}^{2}+{\left(yV^{*}\right)}^{2}+{2|V|}^{2}\left(\right|y^{2}\left|-1\right))\mathrm{snr}+O\left({\mathrm{snr}}^{3/2}\right)\right].$$

Using this Taylor series expansion, the functions $g_{0}\left(y;\mathrm{snr}\right)$ and $g_{1}\left(y;\mathrm{snr}\right)$ defined in (A6) and (A7), respectively, can be computed in terms of the moments of *V* as:

(A11) $$\begin{array}{cc} g_{0}\left(y;\mathrm{snr}\right) & =\psi \left(y\right)\left[1+\frac{1}{2}\left({\left(y^{*}\right)}^{2}E\left[V^{2}\right]+y^{2}E{\left[V^{2}\right]}^{*}+{2E[|V|}^{2}](|y^{2}\left|-1\right)\right)\mathrm{snr}\right], \end{array}$$

(A12) $$\begin{array}{cc} g_{1}\left(y;\mathrm{snr}\right) & =\psi \left(y\right)\left[\left(y^{*}E\left[V^{2}\right]+{yE[|V|}^{2}]\right)\sqrt{\mathrm{snr}}+\frac{1}{2}{\left(y^{*}\right)}^{2}E\left[V^{3}\right]\mathrm{snr}\right], \end{array}$$

where in (A12), we have used $E[|V{|}^{2}V^{*}]=0$. We also have the expansion:

(A13) $$\frac{1}{g_{0}\left(y;\mathrm{snr}\right)}=\frac{1}{\psi (y)}\left[1-\frac{1}{2}\left({\left(y^{*}\right)}^{2}E\left[V^{2}\right]+y^{2}E{\left[V^{2}\right]}^{*}+{2E[|V|}^{2}](|y^{2}\left|-1\right)\right)\mathrm{snr}\right].$$

We are assuming $|E\left[V^{2}\right]{|}^{2}=1$, and in addition, it is easy to check that $E[V^{3}]=0$. Therefore, substituting these values in (A12) and (A13), we get:

(A14) $$g_{1}\left(y;\mathrm{snr}\right)=\psi \left(y\right)\left(y^{*}E\left[V^{2}\right]+y\right)\sqrt{\mathrm{snr}}$$

and:

(A15) $$\frac{1}{g_{0}\left(y;\mathrm{snr}\right)}=\frac{1}{\psi (y)}\left[1-\frac{1}{2}\left({\left(y^{*}\right)}^{2}E\left[V^{2}\right]+y^{2}E{\left[V^{2}\right]}^{*}+2(|y^{2}\left|-1\right)\right)\mathrm{snr}\right],$$

which only depend on $E[V^{2}]$.

Substituting (A14) and (A15) in (A9) and evaluating the resulting integral over *y*, we obtain the result:

(A16) $$\mathrm{mmse}\left(\mathrm{snr},\kappa \right)=1-\left(1+\kappa^{2}\right)\mathrm{snr}+\left(1-\kappa^{2}\right){\mathrm{snr}}^{2}+O\left({\mathrm{snr}}^{3}\right).$$

Finally, by integrating Expression (A16), we obtain the expansion around snr=0 of the mutual information as:

(A17) $$I\left(\mathrm{snr},\kappa \right)=\mathrm{snr}-\frac{\left(1+\kappa^{2}\right)}{2}{\mathrm{snr}}^{2}+\frac{\left(1-\kappa^{2}\right)}{3}{\mathrm{snr}}^{3}+O\left({\mathrm{snr}}^{4}\right).$$

which completes the proof.
